# Toxic Effect of Vancomycin on Viability and Functionality of Different Cells Involved in Tissue Regeneration

**DOI:** 10.3390/antibiotics9050238

**Published:** 2020-05-08

**Authors:** Joy Braun, Stefanie Eckes, Pol Maria Rommens, Katja Schmitz, Daniela Nickel, Ulrike Ritz

**Affiliations:** 1 Department of Orthopaedics and Traumatology, BiomaTiCS, University Medical Center, Johannes Gutenberg University, Langenbeckstraße 1, 55131 Mainz, Germany; Joybraun@uni-mainz.de (J.B.); Pol.rommens@unimedizin-mainz.de (P.M.R.); 2 Clements-Schöpf-Institute of Organic Chemistry and Biochemistry, Technische Universität Darmstadt, Alarich-Weiss-Straße 4, 64287 Darmstadt, Germany; Eckes@biochemie.tu-darmstadt.de (S.E.); Schmitz@biochemie.tu-darmstadt.de (K.S.); 3 Berufsakademie-Sachsen—Staatliche Studienakademie Glauchau, University of Cooperative Education, Kopernikusstraße 51, 08371 Glauchau, Germany; Nickel@ba-glauchau.de

**Keywords:** vancomycin, cell proliferation, cell differentiation, tissue regeneration, local antibiotics

## Abstract

To prevent infections local delivery of antibiotics is a useful tool. Especially in bone fractures, vancomycin impregnated bone cements are often used allowing high concentrations of antibiotics at the infection side without high serum concentrations. However, besides potential pathogens, cells involved in tissue regeneration may also be affected by the drug. We investigated the effect of vancomycin on the viability and functionality on osteoblasts, endothelial cells, fibroblasts and skeletal muscle cells. Our results show that the viability of all cells analyzed was reduced by vancomycin and that the observed effects were time and concentration dependent. The most pronounced toxic effect was detected on day three when even the lowest concentration of 0.01 mg/ml led to a significant decrease in proliferation compared to control. Functionality assays of osteoblasts and skeletal muscle cells revealed a sensitive reaction of the cells to the drug, indicating that vancomycin is toxic to these cells during the process of differentiation. These data suggest that the vancomycin administration is critical for cell survival and function. Therefore, the concentration of administered antibiotics needs to be carefully evaluated to find a balance between defense against pathogens and functionality of host cells and tissues.

## 1. Introduction

Surgical site infections (SSI) are a widespread problem in the field of traumatology and orthopedics [1]. They are defined as microbial contamination of the surgical wound within 30 days after an operation or within one year after surgery when an implant was placed. [2,3]. Organisms responsible for SSI are *coagulase-negative staphylococci*, *Enterococcus spp*., *Escherichia coli, Pseudomonas aeruginosa* and *Enterobacter spp*. [4]. The gram positive *Staphylococcus aureus* is responsible for 15–20% of the SSI occurring in hospitals; thus it is the most commonly isolated organism in SSI [4]. SSI cause pain and stress for patients as well as additional health costs [5]. The incidence of such infections depends not only on the procedure but also on the surgical site. For example the infection rate after major allograft bone transplantation can be as high as 13% [6], the chance of infection in transcutaneous fracture pins lies in a range of 2–30% [7,8,9,10] and the rate of infection after spinal surgeries is 2–5% [11,12]. To prevent this, the prophylactic administration of antibiotics right before the surgery is a common tool, however, combined with lots of potential disadvantages. In systemic administration the drug concentration at the target site is often very low. Furthermore the correct time of administration plays an important role [13]. Often the antibiotic needs to be administered in high dosages for a long time period which can cause systemic side effects. Especially in the orthopedic field where septic diseases like osteomyelitis are likely to occur the local delivery of antibiotic agents is a good alternative. Polymethylmethacrylate (PMMA) bone cements with antibiotics or implants with antibiotic-loaded coatings on their surface are often used [14,15]. Such drug delivery systems enable the local release of drugs in order to achieve high concentrations on the potential infection site without causing high serum concentrations [16,17]. Thus, it is possible to reduce systemic toxicity as well as bacterial resistance to antibiotics [18]. Recommendations and guidelines for prophylactic administration of antibiotics exist but there are no guidelines for the administration of antibiotics to the local site for example by means of bone cements enhanced with antibiotics [12,19,20,21]. Which antibiotic is used in which concentration depends not only on the surgeon, clinical evidence and manufacturer’s instructions, but also on the expected germs. Further, the type of antibiotic as well as its quantity depend on the patient’s individual medical history (e.g., diabetes), body weight and potential allergies. Due to the above mentioned points, the concentration of antibiotic at the site of injury varies greatly. Especially in local therapies where the concentration of the antibiotic is often very high, it seems to be important to know how the surrounding tissues and cells are affected by the drug.

In bone cements aminoglycosides like gentamycin and vancomycin, antibiotics of the glycopeptide group, are commonly used [22]. They fulfill the requirements for the use in such cements like thermal stability, availability in powder form and low serum protein binding as well as a focused spectrum of activity and being inexpensive [23,24]. Only few reports exist analyzing the influence of antibiotics on viability, morphology and other parameters of different cell types like human corneal endothelial cells, murine skeletal muscle cells (C_2_C_12_), human umbilical vein endothelial cells (HUVECs), osteoblasts, bone marrow-derived mesenchymal stem cells (bmMSC) and the human osteosarcoma cell line (MG-63) [16,25,26,27,28,29]. To our knowledge there is no publication describing the effect of antibiotics to all common human and primary cell types involved in tissue and bone regeneration. Since our focus is on bone regeneration and vancomycin is one of the four most frequently used antibiotics in orthopedics and trauma surgery [30], we investigated the influence of this drug on cell proliferation, differentiation and functionality on human (primary) osteoblasts, endothelial cells, muscle cells and fibroblasts. Vancomycin was chosen because of its broad application area in surgery for the prevention of infections [31,32,33,34,35]. For example it is a potent drug to eliminate pathogens like the above mentioned *Staphyloccous aureus* which is the most isolated bacteria in SSI [4,36].

## 2. Results

### 2.1. Micobiology (Determination of the Minimal Inhibitory Concentration (MIC))

**Figure 1:**
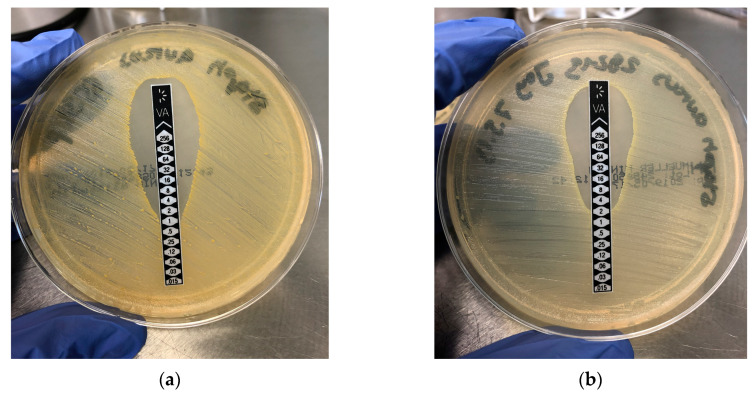
Determination of vancomycin MIC (µg/mL) with *Staphylococcus aureus* 6538P (**a**) and *Staphylococcus aureus* 29213 (**b**).

To determine the concentrations of Vancomycin to be used for cell testing a MIC test of the drug on two different *Staphylococcus aureus* strains was made. The MIC for the *Staphylococcus aureus 6538P* was 1 µg/mL (Figure 1a) and the MIC for the second strain *29,213* was 2 µg/mL (Figure 1b). As the environment of the body is more complex (blood flow, enzymes etc.) than in a petri dish, we decided to perform our cell experiments with concentrations ranging from 0.01 mg/mL to 2mg/mL.

### 2.2. Cell Viability

**Figure 2:**
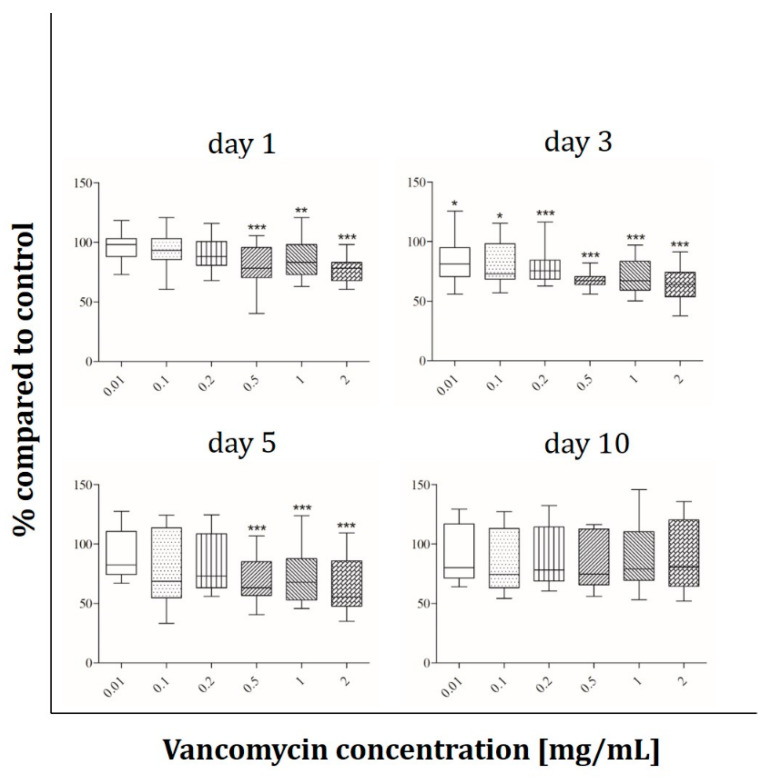
Proliferation of primary human muscle cells after exposure to vancomycin for 1, 3, 5 and 10 days with change of antibiotic every four days (* *p* ˂ 0.05; ** *p* < 0.01; *** *p* < 0.001 to control). The results are presented in percentage of the control without vancomycin (100%).

Cell viability under the influence of vancomycin (0.01, 0.1, 0.2, 0.5, 1 and 2 mg/mL) was tested with the alamarBlue^®^ Assay on days 1, 3, 5 and 10 after starting the incubation period. The results are presented in percentage of the control without vancomycin (100%). The most sensitive cells were the human skeletal muscle cells (Figure 2). On day one a significant decrease in cell viability of around 20% was observed at concentrations starting from 0.5 mg/ml. Muscle cells reacted more sensitive on day three when a reduction of 15% in cell viability could already be detected with the lowest vancomycin concentration of 0.01 mg/mL. Cell viability was reduced up to 37% at the highest concentration (2 mg/mL) when compared with the control group without vancomycin. After five days the same effects as on day one were observed, however on the last day of measurement no significant difference in viability compared to control was observed.

**Figure 3:**
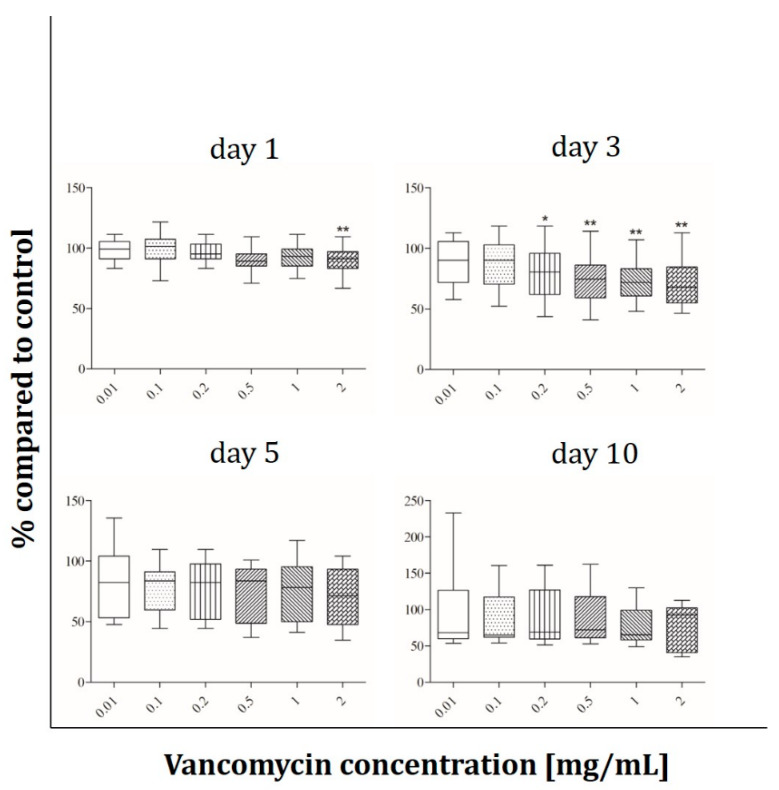
Proliferation of human osteoblasts after exposure to vancomycin for 1, 3, 5 and 10 days with change of antibiotic every 4 days (* *p* ˂ 0.05; ** *p* < 0.01; *** *p* < 0.001 to control). The results are presented in percentage of the control without vancomycin (100%).

Human primary osteoblasts (Figure 3) show a significant decrease of proliferation on day one only at the highest vancomycin concentration whereas they react more sensitive after three days of incubation. There was a range of 20% to 30% of cell viability reduction with increasing concentration up from 0.2 mg/mL. On the third and fourth time points no significant inhibition of proliferation compared to the untreated cells was detected.

**Figure 4:**
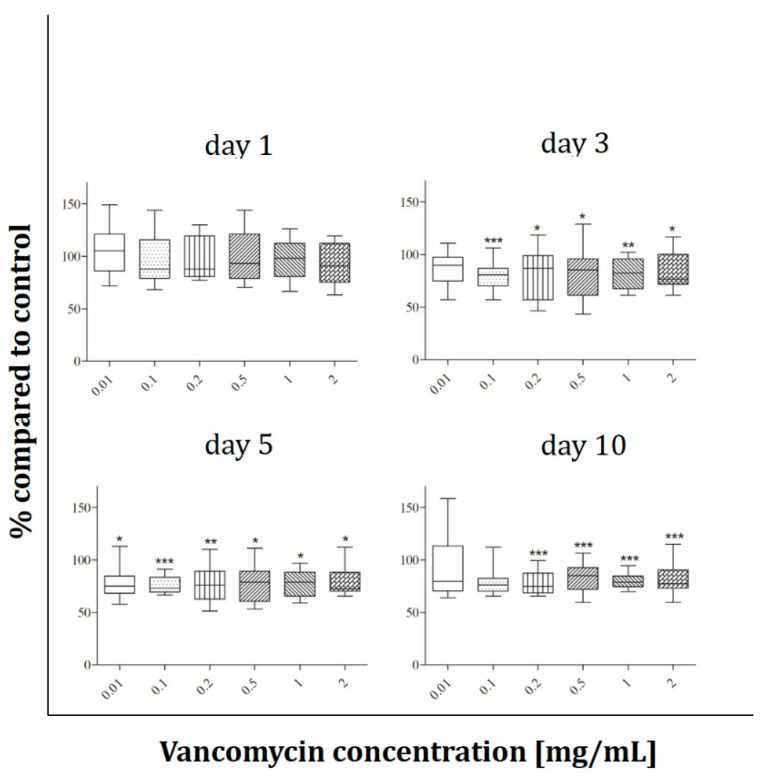
Proliferation of Saos-2 cells after exposure to vancomycin for 1, 3, 5 and 10 days with change of antibiotic every 4 days (* *p* ˂ 0.05; ** *p* < 0.01; *** *p* < 0.001 to control). The results are presented in percentage of the control without vancomycin (100%).

The osteoblast-like cell line Saos-2 behaves different than the primary osteoblasts. In contrast to hOBs no influence of vancomycin after 24 hours of incubation was detected while the Saos-2 cells reacted more sensitive on day three with an approximately 20% lower viability starting from 0.1 mg/mL vancomycin (Figure 4). After five days every used concentration affected the viability of the cells but the extent of decrease was not affected by the duration of incubation. At the last time point the viability increased compared to days three and five, but a decrease in cell viability was still observed when collated to the untreated control.

**Figure 5:**
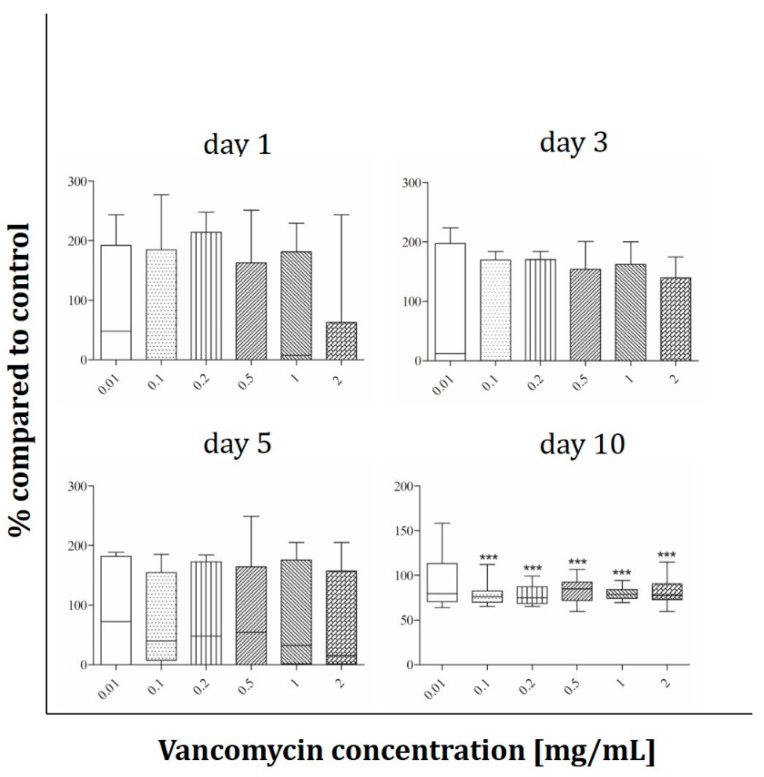
Proliferation of HUVECs after exposure to vancomycin for 1, 3, 5 and 10 days with change of antibiotic every 4 days (* *p* ˂ 0.05; ** *p* < 0.01; *** *p* < 0.001 to control). The results are presented in percentage of the control without vancomycin (100%).

The viability of HUVECs was not affected by vancomycin until day five, while the cells reacted very sensitive after 10 days (Figure 5). Only the smallest concentration showed no negative effect on the viability; in all other concentrations a decrease was detected in the range between 23% and 44%.

**Figure 6:**
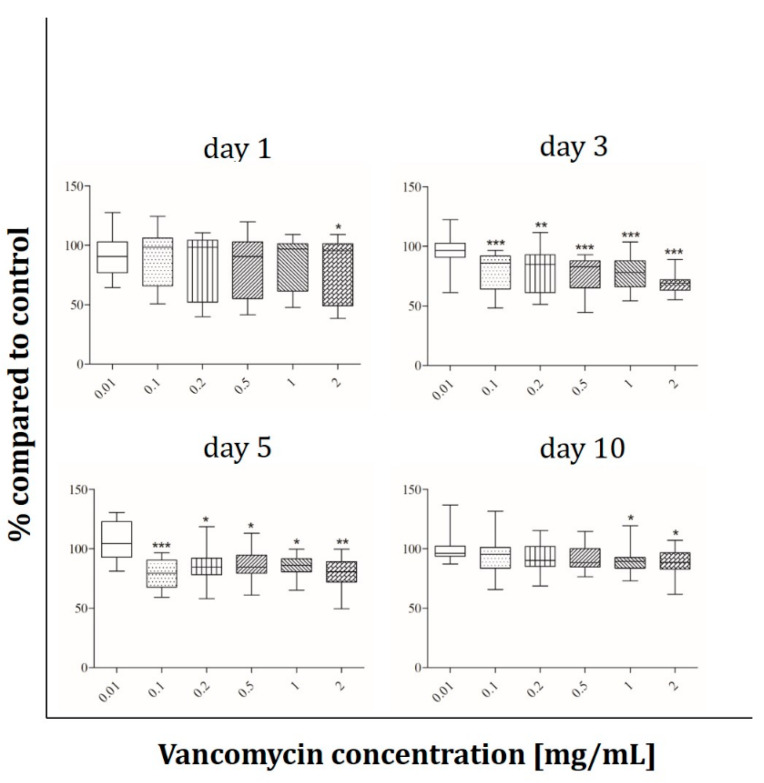
Proliferation of fibroblasts after exposure to vancomycin for 1, 3, 5 and 10 days with change of antibiotic every 4 days (* *p* ˂ 0.05; ** *p* < 0.01; *** *p* < 0.001 to control). The results are presented in percentage of the control without vancomycin (100%).

Fibroblasts behave like osteoblasts within the first 24 hours with only 80% of viability at the highest concentration compared to control (Figure 6). A significant decrease in proliferation was detected for 0.1 mg/mL vancomycin and above on day three and five from 20% to 30% on day five. At the last time point the reaction of the cells was less pronounced, however at concentrations of 1 mg/mL and above viability was still significantly reduced.

### 2.3. Angiogenesis Assay

**Figure 7:**
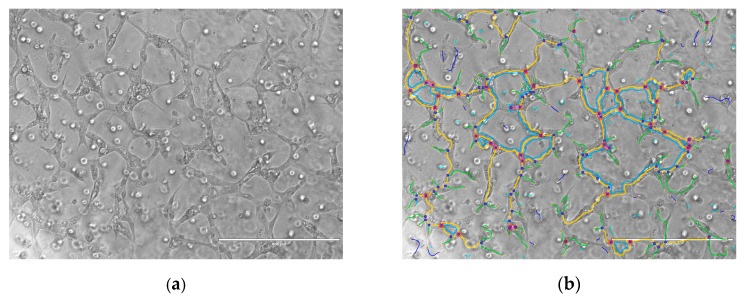
Example of an angiogenesis assay of HUVECs after exposure to 0.1 mg/mL vancomycin. Microscopic image of tube formation in Matrigel (**a**) and evaluation with the angiogenesis analyzer for ImageJ by Gilles Carpentier (2012) where different colors represent different parameters (**b**).

To test whether vancomycin induces or inhibits angiogenesis, HUVECs were exposed to different vancomycin doses (0.01–2 mg/mL) in Matrigel. Images were analyzed with the angiogenesis analyzer for ImageJ by Gilles Carpentier (2012) (Figure 7).

**Figure 8:**
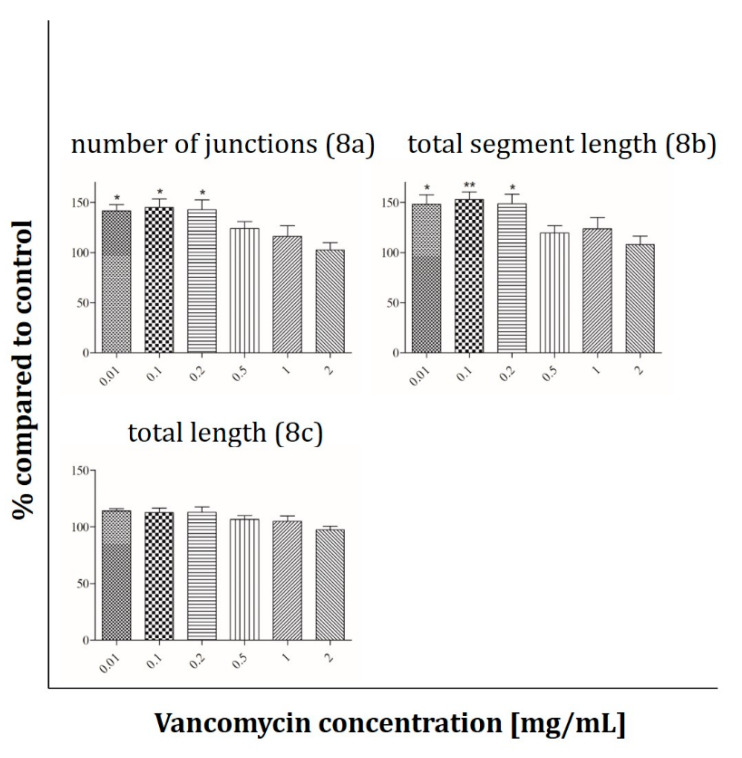
Tube formation assay of HUVEVs after exposure to vancomycin. Parameters used for evaluation of the results were: number of junctions (**a**), total segment length (**b**) and total length (**c**) (* *p* ˂ 0.05; ** *p* < 0.01; *** *p* < 0.001 to control. The results are presented in percentage of the control without vancomycin (100%).

To quantify the results the following parameters were used: total length, number of junctions and total segment length. Compared to control without vancomycin administration a significant increase in the number of junctions and total segment length was observed at low vancomycin concentrations in the range from 0.01–0.2 mg/mL (Figure 8a,b). Comparing both parameters angiogenesis was up to 40–50% higher than in cells treated with culture media without drug. However, the total length of the tube formation by HUVECs was not influenced either in low or in high antibiotic concentrations (Figure 8c).

### 2.4. Immunofluorescence Analysis

**Figure 9:**
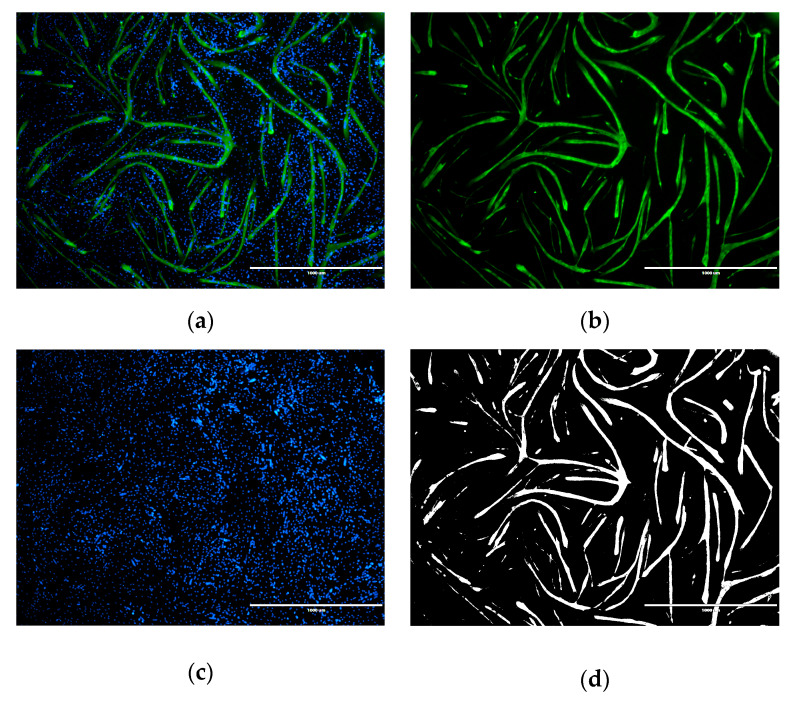
Immunofluorescence analysis of skeletal muscle cells differentiated for 5 days. Microscopic images: overlay (**a**) of the myosin staining (**b**) and the nuclei staining (**c**). To quantify the influence of vancomycin on myotube formation the binary image (**d**) was used.

Immunofluorescence was performed to detect the influence of vancomycin on the differentiation of human muscle cells. Muscle marker protein myosin was used for the detection of myotube formation and the total area was measured (Figure 9) [37].

**Figure 10:**
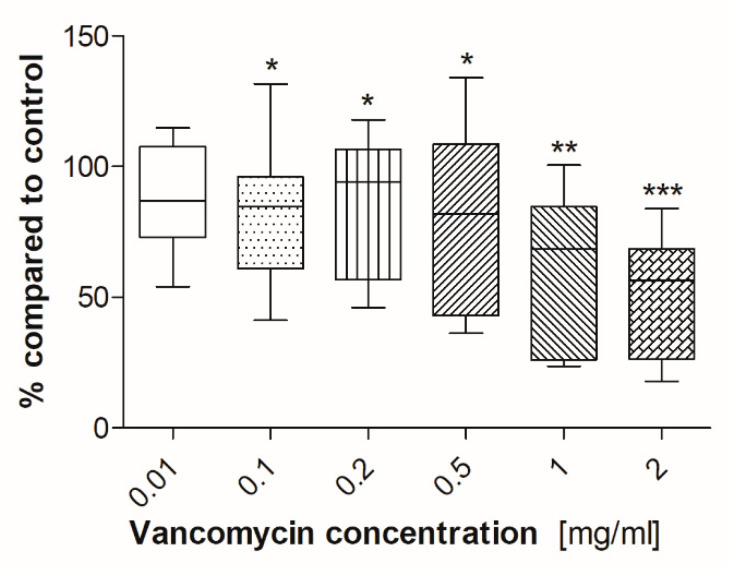
Myotube formation of human muscle cells after exposure to vancomycin (* *p* ˂ 0.05; ** *p* < 0.01; *** *p* < 0.001 to control). The results are presented in percentage of the control without vancomycin (100%).

There was a significant inhibition of myotube formation after incubation of cells with vancomycin at concentrations of 0.1 mg/mL and above (Figure 10).

### 2.5. ALP Activity

**Figure 11:**
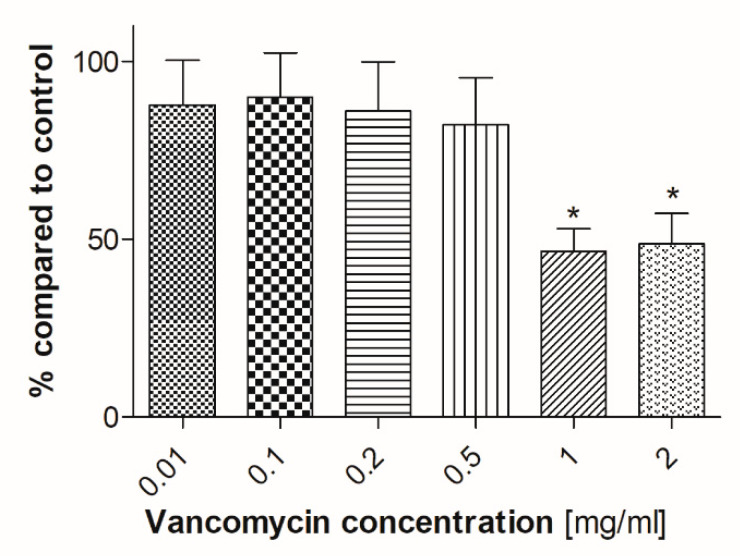
ALP activity of human osteoblasts after exposure to vancomycin for 5 days (* *p* ˂ 0.05; ** *p* < 0.01; *** *p* < 0.001 to control). The results are presented in percentage of the control without vancomycin (100%).

ALP is a specific marker during the early stage of osteogenic differentiation [38]. Therefore, the ALP assay was conducted to evaluate if vancomycin affects the osteogenic differentiation in hOB after five days (Figure 11). While there was no significant difference in ALP expression level in cells incubated with vancomycin in a range from 0.01–0.5 mg/mL, the ALP level decreased statistically significant at high concentrations (1–2 mg/mL). Compared to the control the ALP activity was diminished by over 50% (1 mg/mL, 46.61%; 2 mg/mL, 48.69%) in osteoblasts incubated at high concentrations of vancomycin.

## 3. Discussion

The prophylactic and therapeutic local delivery of antibiotics is a common tool to achieve maximum impact at the site of infection without systemic toxicity. Especially in the field of traumatology and orthopedics, bone cements or implants impregnated with antibiotics are often used to prevent or treat infections. This delivery method allows high doses in local tissue in different time frames. It is recommended to use 3.6 mg of antibiotic in 40 mg of acylic bone cement to guarantee the optimal drug level during therapy of existing infections. If the drug is used for prophylaxis a much lower concentration of only 1 mg/ml is needed [39,40,41]. Nevertheless these are only recommendations and often the drugs are applied in higher doses or in combination [42,43,44,45]. In trauma surgery and orthopedics the glycopeptide antibiotic vancomycin is often administered [31,46,47]. It is an effective antibiotic and kills bacteria by hindering cell wall synthesis. However, to what extent different concentrations influence the surrounding host tissues and the cells involved in tissue and bone regeneration is not well explored and discussed in the literature. There are only a few studies investigating the influence of different antibiotics on cells and these studies mostly used cell lines, sometimes only murine cell lines, which behave different than primary human cells. Rathbone et al. investigated the influence of 21 antibiotics in a concentration range from 0–5 mg/mL on cell viability and ALP activity of human primary osteoblasts with the result that vancomycin apart from amikacin and tobramycin was the least cytotoxic one. Only in very high concentrations of 5 mg/mL the cell number as well as the ALP activity decreased [16]. Another study showed that concentrations of vancomycin up to 1 mg/mL had no effect on cell viability of the osteoblastic-like cell line MG63, whereas concentrations of 10 mg/mL caused cell death [29]. The effect of vancomycin on cell viability of HUVECs was tested by Drouet et al. They could show that the toxicity of vancomycin is concentration- and time-dependent with a negative effect on proliferation after 24 hours at a concentrations of 5 and 2.5 mg/mL after 72 h [27]. Nevertheless, to our knowledge no study has been reported analyzing the influence of vancomycin on proliferation, differentiation and functionality of various human cells involved in tissue/bone regeneration. We investigated the effect of vancomycin on viability and functionality of human cells lines as well as of human primary cells which are involved in tissue regeneration and bone healing, in particular. These included: osteoblasts (primary cells and cell line), endothelial cells, primary skeletal muscle cells and primary fibroblasts. To evaluate the toxicity of vancomycin on proliferation the alamarBlue^®^ assay was used.

### 3.1. Microbiology (MIC Determination)

The MIC-test was used to determine the Vancomycin concentration necessary to inhibit bacteria as well as the concentration needed for testing Vancomycin effects in cellular experiments. Therefore, two *Staphylococcus aureus* strains were used which were isolated from a lesion respectively a wound. The lowest concentration of Vancomycin which inhibited the visible growth of the strains was 1 and 2 µg/mL, respectively. This indicated that (especially in the *Staphylococcus aureus* strains which we used) a very small concentration is needed to kill the bacteria. As comparison bone cements are loaded with 1 mg/ml of Vancomycin [39,40,41].

### 3.2. Cell Viability

The results showed that every single cell type reacted in a different way and that the inhibition of cell growth was not only concentration but also time dependent. We observed a significant inhibition of fibroblast cell proliferation after three and five days of incubation using low concentrations of vancomycin. Only at the last time point (10 days after starting the incubation) the cells seemed to recover when low drug concentrations were used. However, concentrations higher than 1 mg/mL were still toxic over the entire time period tested. Primary skeletal muscle cells exhibited the highest sensitivity on day three with a reduction in viability in every used concentration. Moreover an inhibition was detected at 0.5 mg/mL and above on days one and five while no significant differences were measured on day 10 indicating a similar recovery as in the primary skeletal muscle cells. Whereas hOB showed no toxicity on days five and ten, the osteoblast-like cell line Saos-2 reacted more sensitive on day five with decreasing proliferation even in the smallest concentration of 0.01 mg/mL compared to control. These different results highlight the importance to perform such experiments not only with cell lines but in particular with primary cells. Statistically there were no significant differences in viability of HUVECs within the first three time points. Experiments were performed independently three times and different results were observed resulting in a rather high standard deviation. For this reason the results of the HUVECs are difficult to analyze and it has to be kept in mind that these are very sensitive primary cells. In general the lowest toxicity was detected after one day of incubation with vancomycin. All cells (apart from HUVECs) showed increased sensitivity to vancomycin on day three compared to day one. Generally, we observed a reduced sensitivity towards vancomycin from day five to day 10 in all used cell types except HUVECs and an increase in sensitivity from day one to day three.

The reduction of proliferation which was detected was therefore not only dose but also time dependent. This indicates that an initial burst release over the first 24 h hours would be better for the surrounding tissue than a steady state over a longer time period. Compared to the published studies our results show a more sensitive reaction to vancomycin in all tested cell types. We could detect cytotoxic effects of the drug in much lower concentrations than 5 mg/mL as described by Rathbone [16]. Every single cell type showed at least at one time point a significant inhibition in proliferation at a concentration of 2 mg/mL. Moreover, in all experiments, a significant decrease in cell viability was detected even at low concentrations. The study by Rathbone et al. is the only one including primary hOB. They tested the cells 10 and 14 day after the incubation period started and postulated that vancomycin is one of the least toxic antibiotics. However, they did not consider the first days. In agreement with this study we detected no toxicity on day 10 in our hOB experiments at 2 mg/mL. In contrast to Rathbone’s study, our viability results showed that vancomycin is cytotoxic even in small concentrations, therefore we disagree with the assumption that vancomycin is a very low toxic antibiotic.

Another interesting result from this study is that most of the cells showed an increase in cell viability from day 5 to 10. This indicates that the cells may habituate to the drug. Since vancomycin was changed every four days, it is unlikely that vancomycin was metabolized by the cells. To explain this cell behavior more research needs to be done in this field.

### 3.3. Cell Functionality

To clarify our results we performed some experiments concerning the functionality of the used primary cells. To our knowledge no studies exist analyzing the effect of vancomycin on functionality of different cell types. Therefore, the influence of vancomycin on myotube formation of muscle cells, ALP activity of osteoblasts and tube formation of HUVECs was examined. For the functionality test of muscle cells and osteoblasts, cells were incubated in the respective differentiation medium supplemented with vancomycin for five or in case of osteoblasts for six days. Muscle cells showed an inhibition of myotube formation at low concentrations of 0.1 mg/mL and above whereas, during proliferation, the limit of toxicity was detected at a vancomycin concentration of 0.5 mg/mL. On osteoblasts no significant differences were measured in viability on day five, but a reduced ALP activity could be shown after incubation in differentiation medium with 1 mg/mL or higher vancomycin concentrations. Compared to the proliferation results on the respective days the cells reacted more sensitive to the antibiotic during differentiation. The influence of vancomycin on the functionality of endothelial cells is not only interesting for the regeneration of tissue but also for some other clinical applications. With a pH of less than 4, vancomycin is classified as very acidic. To overcome the problem of vein irritation the drug is administered by infusion into the central venous catheter and therefore it gets in close contact with this type of cells [48]. Additionally, an inhibition of tube formation could be interesting for tumor therapy. It was shown that, for example, clarithromycin is a potent inhibitor in tumor-induced angiogenesis [49]. To analyze the influence of the drug on the tube formation of HUVECs three parameters were used: number of junctions, total segment length and the total area. A positive effect was detected on the first two mentioned parameters in small concentrations in the range from 0.01–0.2 mg/mL compared to control and higher doses. Nevertheless no positive effect was measured on the total length. To date the effect of antibiotics on the function and differentiation pathways of the used cells still needs to be elucidated.

## 4. Conclusion

Although our study shows that even small concentrations of vancomycin have a negative effect on proliferation and functionalization of various cell types, our in vitro study does only partially reflect conditions in vivo. In cell culture there is a completely different environment including tissue flow, serum as well as enzyme presence compared to applications after an injury in humans. Still, our results indicate that, in the clinical practice the amount of administered antibiotic should be reconsidered and optimized. It should be kept in mind that high concentrations cannot only prevent infections, but could also inhibit or alter tissue regeneration. More detailed analyses should be performed to evaluate the right balance between antibacterial defense and influence on physiological tissue.

## 5. Materials and Methods

### 5.1. Microbiology (MIC Determination)

Since the Gram-positive *Staphylococcus aureus* is the most commonly isolated organism in SSI it was chosen to determine the MIC. Therefore Culti-Loops™ *Staphylococcus aureus* subsp. *aureus* ATCC™ 29213™ (isolated from a lesion) and Culti-Loops™ *Staphylococcus aureus* subsp. *aureus* ATCC^®^ 6538P™ (isolated from a wound) (Fisher scientific GmbH, Schwerte, Germany) were solved in 2 mL NaCl solution. Before MIC experiments, an overnight culture was performed on Trypticase™ Soy Agar (Fisher scientific GmbH, Schwerte, Germany) at 37 °C. For the MIC testing individual colonies from the stock culture were picked up, diluted in 1 mL NaCl and compared to a 0.5 McFarland Standard (Carl Roth, Karlsruhe, Germany). A smear preparation was made on Mueller Hinton Agar and a vancomycin test stripe with a concentration gradient of 256–0.016 µg/mL (Oxoid Deutschland GmbH, Wesel, Germany) were prepared on the middle. After 24 h of incubation at 37 °C the MIC were determined. Since the vancomycin concentrations determined in the MIC-tests are very small and as the environment of the body is more complex (blood flow, enzymes etc.) than in a petri dish, we decided to start our cell experiments with a 10× higher concentration of 0.01 mg/mL. As the highest concentration in our in vitro experiments, we chose a concentration 2000 times higher than the MIC (2 mg/mL).

### 5.2. Vancomycin

Vancomycin stock solutions (Carl Roth, Karlsruhe, Germany) were prepared in distilled water (100 mg/mL and 10 mg/mL). Final concentrations of 0.01, 0.1, 0.2, 0.5, 1 and 2 mg/mL were prepared by dilutions in the respective cell culture medium without penicillin and streptomycin.

### 5.3. Cell Culture

Normal human dermal fibroblasts (NHDF; PromoCell, Heidelberg, Germany) and *Homo sapiens* bone osteosarcoma cells (Saos-2; ATCC, Manassas, VA, USA) were maintained in Dulbecco’s’ Modified Eagle Medium + GlutaMAX (Gibco, Life Technologies, Grand Island, NY, USA) containing 10% fetal calf serum (FCS) (Biochrom AG, Berlin, Germany) and 100 U/mL penicillin, 100 μg/mL streptomycin sulphate (Sigma-Aldrich^®^GmbH, St. Louis, MO, USA). Primary cultures of human umbilical vein endothelial cells (HUVEC) were purchased from PromoCell (Heidelberg, Germany), cultured in endothelial cell growth medium MV (PromoCell, Heidelberg, Germany) and supplemented with the reagents provided by the kit.

Bone fragments from hip replacements of three different patients were used for isolation of primary human osteoblasts (hOBs). Informed consent was obtained from all patients and the local ethics committee approved the investigations. Isolation of human osteoblasts followed a previously described protocol [50,51]. Briefly, attached fibrous and fat tissue was carefully removed from bone specimen. Fragments were vigorously rinsed in phosphate-buffered saline (PBS) and digested with collagenase type IV (Sigma Aldrich^®^ GmbH, St. Louis, MO, USA) for 45 min at 37 °C. After washing in PBS to remove residual blood and fat, three ∼2 mm^3^ bone specimens were placed in 6-well plates (Becton-Dickinson, Heidelberg, Germany) and cultured in DMEM/F12 (Biochrom, Berlin, Germany) supplemented with 10% FCS (PAA Lab, Austria), 100 U/mL penicillin, 100 μg/mL streptomycin sulphate. For differentiation the medium was additionally supplemented with 100 μg/mL ascorbate, 50 μg/mL glycerolphosphate, and 10^−8^ M dexamethasone and the cells were incubated in a humidified atmosphere (5% CO_2_, 37 °C, medium changed twice a week). For isolation of muscle cells tissue from the lumbar, columns of three different donors were used. Informed consent was obtained from all patients and the local ethics committee approved the investigations. The cultivation of the cells took place in cell culture bottles coated with collagen (Collagen type 1 (CORNING^®^, Discovery Labware, Amsterdam, Netherlands)/PBS (1:100 (v:v)), 45 min incubation). After removal of perimysium, cell debris as well as unspecific tissue the muscle samples were hackled in 1 mm^2^ fragments. Afterwards the tissue was washed and incubated in collagenase (type 2, 470 U/mL, Worthington Biochemical Corporation, Lakewood, CO, USA) in DMEM/F-12) at 37 °C in a water bath. After 1 h samples were centrifuged (7 min, 1.600 rpm), the supernatant was discarded and a second period of incubation with trypsin/EDTA (0.25%/0.02%, 20 min; Biochrom GmbH, Berlin) was started. Trypsination was stopped by adding medium and samples were filtered with a cell filter followed by a centrifugation step (1400 rpm, 5 min). The pellet was resuspended in media and the suspension was cultured for 2 h in the incubator before the medium-containing muscle cells were transferred into another cell culture flask.

### 5.4. Viability and functionality assays

#### 5.4.1. AlamarBlue^®^ Assay

Cell viability and proliferation were tested with the alamarBlue^®^ assay. The non-cytotoxic reagent allows repeated measurements at different time points. For the test 2500 cells/well were seeded in a 48-well plate and incubated in medium. After 24 hours the medium was replaced by medium containing vancomycin in different concentrations, every four days the medium was changed. The alamarBlue^®^ assay (Gibco^®^Invitrogen™ Life Technologies, Carlsbad, CA, USA) was performed 1, 3, 5 and 10 days after the beginning of the vancomycin incubation. For this purpose, the vancomycin solution was removed and the cells were incubated with 320 µL of a 10% solution of alamarBlue^®^ in cell culture medium for 4 h at 37 °C. Subsequently 3 × 100 µL of the supernatant were transferred into a 96-well plate and the absorbance (560/600nm) was measured.

#### 5.4.2. Angiogenesis Assay

To examine the effect of vancomycin on in vitro angiogenesis Matrigel was used. HUVECs (passage 3–5; 5 × 10^4^ cells/well) were diluted in Matrigel (Beck Dickinson, Heidelberg, Germany) 1:2 and 50 µL of the mixture were filled into a 96-well plate. For polymerization the plate was placed in the incubator for 30 min at 37 °C followed by addition of vancomycin. The cells were incubated in the drug containing medium for 24 h before they were analyzed with the EVOS^®^ digital Inverted Microscope (Life Technologies, Carlsbad, CA, USA).

#### 5.4.3. Immunofluorescence Analyses

Immunofluorescence staining was used to determine the effect of vancomycin on the differentiation of the isolated muscle cells. Therefore cells were cultured in collagen (Corning^®^; Discovery Labware; Amsterdam; Netherlands) coated 24 well plates. After reaching 80% confluency the medium was replaced by differentiation medium including vancomycin in different concentrations. Five days later, cells were washed twice with PBS, followed by fixation with ice cold methanol (Carl-Roth^®^ GmbH, Karlsruhe, Germany) for 20 min. After a second washing step with PBS and 20 minutes incubation with the primary antibody skeletal muscle myosin (F59) anti mouse (1:200 in 0,5 % BSA/PBS; Santa Cruz Biotechnology, Texas, TX., USA) was added and incubated at 4 °C overnight. As secondary antibody Alexa 488 goat anti mouse IgG (1:200; Invitrogen™Life Technologies, Carlsbad, USA) was applied for 1 h in the dark followed by washing with PBS. Finally nuclei were stained with Hoechst (4 µg/mL; Sigma Aldrich, St. Louis, MO, USA) with an incubation time of 15 minutes at room temperature in the dark. Detection was performed with the EVOS^®^ digital Inverted Microscope (Life Technologies, Carlsbad, CA, USA).

#### 5.4.4. ALP Activity

Alkaline phosphatase (ALP) assay was used to determine the osteoblast mineralization capacity during treatment with various concentrations of vancomycin. Human osteoblasts were seeded in 24-well plates and incubated in growth medium until confluence at 37 °C with 5% CO_2_. Growth medium was replaced by differentiation medium including vancomycin and the cells were incubated for six days with media change on day four. For the ALP assay cells were washed twice with PBS and were then incubated for 30 min, at 37 °C with 250 µL of 0.1% Triton™ X-100 (Sigma-Aldrich^®^GmbH, St. Louis, MO, USA) in aqua bidest. Afterwards 200 µL of p-nitrophenylphosphate (Sigma-Aldrich^®^GmbH, St. Louis, MO, USA) were added to the cell culture well and another incubation period of 30 minutes was started. To stop the reaction 50 µL of 5 M NaOH (AppliChem GmbH, Darmstadt, Germany) were added to each well and 100 µL per sample were transferred to a 96-well plate. The absorbance was measured at 405/700 nm and a calibration curve was used to determine the final concentration of ALP.

### 5.5. Statistical Analysis

Statistical analysis was conducted with the SPSS software (Version 24). Values are presented as means ± SD. All experiments were performed independently three times. Tests with primary cells were performed with cells from three different donors in three independent experiments, respectively. For the analysis a one-way analysis of variance (ANOVA) was used. In case of homogeneity of variance (Levene´s test, *p* > 0.05) the Tukey post hoc analysis was performed. Games–Howell post hoc analysis was performed when Levene´s test was *p* < 0.05 and the homogeneity of variance were not given. A *p*-value of <0.05 was assumed as statistically significant.
